# Computer-aided simulation and exergy analysis of TiO_2_ nanoparticles production via green chemistry

**DOI:** 10.7717/peerj.8113

**Published:** 2019-11-25

**Authors:** Samir Meramo-Hurtado, Kariana Moreno-Sader, Ángel D. González-Delgado

**Affiliations:** Nanomaterials and Computer Aided Process Engineering Research Group (NIPAC), Department of Chemical Engineeering, University of Cartagena, Cartagena de Indias, Colombia

**Keywords:** Lemongrass, TiO2 nanoparticles, Exergy analysis, Process simulation, GreenChemistry

## Abstract

**Background:**

The production of photocatalytic nanoparticles such as TiO_2_ has received increasing interest for biomedical and wastewater treatment applications. However, the conventional synthesis of such materials faces several environmental concerns.

**Methods:**

In this work, green synthesis is addressed to prepare TiO_2_ nanoparticles at large scale using Lemongrass (*Cymbopogon citratus*) and titanium isopropoxide (TTIP). This process was designed and modeled using computer-aided process engineering (CAPE) in order to obtain the extended mass/energy balances, as well as operating parameters. Process simulation was carried out using the commercial software Aspen Plus^®^. In addition, energy performance of large-scale nanoparticle production was analyzed to identify alternatives for process improvement from an exergetic point of view.

**Results:**

The production capacity of the plant was estimated as 1,496 t/y of TiO_2_ nanoparticles by the conversion of 32,675 t/y lemongrass and 5,724 t/y TTIP. Hence, the overall production yield is 0.26 kg TiO_2_/kg TTIP. Exergy analysis reported an overall exergy efficiency of 0.27% and an exergy loss of 159,824.80 MJ/h. These results suggest that such a process requires the implementation of process improvement strategies to reach a more sustainable design from energy and thermodynamic viewpoints.

## Introduction

In recent years, several research contributions have been reported about sustainable and green chemistry-based pathways to produce novel materials widely applied in different industries. Among such applications, wastewater treatment is recognized as key issue around the world to preserve the quality of water resource. Moreover, appropriate technologies for wastewater (industrial and domestic) treatment have attached a great interest in order to meet environmental regulations (Bhojwani et al., 2019). On the other hand, advanced oxidation processes are promising alternatives for wastewater treatment purposes since these technologies use photocatalytic reaction systems to degrade pollutants in water under solar or radiation influence (Bustillo-Lecompte, Kakar & Mehrvar, 2018). For such technology, titanium dioxide (TiO_2_) is the most used photocatalyst because of its high efficiency to remove pollutants from water (Acosta-Herazo et al., 2019). Despite the efforts towards more sustainable synthesis of titanium dioxide, there is a knowledge gap in large-scale production of such photocatalytic nanoparticles via green chemistry methods.

Anastas and Warner introduced guidelines to make greener processes and products through twelve design principles of green chemistry, which can be summarized as follows: waste prevention, atom economy, safer synthesis, safer products, safer auxiliaries, energy efficiency, renewable feedstocks, derivative reduction, catalysis, degradability, and pollution prevention (Anastas & Warner, 1998). These principles are mainly focused on reducing the hazard of chemicals and designing products that easily biodegrade after their useful life. Green chemistry is an innovative concept associated with the natural change of pollution prevention towards the synthesis of novel products considering sustainable parameters. Processes meeting green chemistry principles usually employ operations that require less energy consumption, which helps to increase economic profits by reducing operational costs (Hendershot, 2015). Recently, green chemistry concepts were also applied to conversion operations of high-value products to increase process efficiency (Deyris & Grison, 2018).

Nanotechnology is a revolutionary science related to the handling of substances at molecular or atomic level. The development of manufacturing processes from nanotechnology principles is an interesting research topic to design novel pathways for industrial production of nanomaterials (Wang et al., 2016). Several works have been conducted related to the green synthesis and characterization of metal oxide nanoparticles due to their potential application in different fields (Nwankwo et al., 2019). Karpagavinayagam & Vedhi (2019) presented a novel green synthesis of iron oxide nanoparticles using *Avicennia marina* flower extract. Arya, Mishra & Chundawat (2019) developed a green synthesis of silver nanoparticles from *Botryococcus braunii* and evaluated the catalytic behavior of such nanoparticles for benzimodazoles production. This research reveals a growing interest in producing nanoparticles through green chemistry methods for several uses, e.g., green TiO_2_ nanoparticles are commonly used as photocatalyst for pollutant degradation and virus sterilization among other application (Haider et al., 2017).

Scaled-up technologies demand industrial utilities and a water supply that can be reduced by incorporating process improvements towards sustainable practices. Many contributions reported in the literature address the assessment of chemical processes using computer-aided tools, e.g., exergy analysis methods to estimate energy performance. Exergy is defined as the available theoretical work of a system through a process that can be obtained by bringing the system into equilibrium with a heat reservoir or the environment (Martínez González, Silva Lora & Escobar Palacio, 2019). All thermodynamic processes present irreversibilities; hence, the exergy of such systems is not conserved. This is explained by the dissipation of potentially useful energy to generate work. An exergy assessment allows one to identify system components or equipment (of any process) with the highest exergy losses. Another important feature of exergy analysis is the estimation of inefficiencies and identification of the sources responsible for such inefficiencies (Querol, Gonzalez-Regueral & Perez-Benedito, 2013). The first and second laws of thermodynamics are the basis of exergy analysis. These theoretical foundations provide insights about the direction of processes, their irreversibilities, the maximum reversible work, and its thermodynamic efficiency.

To date, limited research literature exists to simulate and evaluate the green synthesis of photocatalytic nanoparticles at large-scale and several contributions are restricted to lab-scale preparation of such nanomaterials. The novelty of this work lies in the scaling-up and exergy assessment of a green chemistry-based process for TiO_2_ nanoparticles to estimate the overall production yield and identify improvement opportunities. Process modeling and simulation of TiO_2_ nanoparticle production is performed through CAPE tools, which requires process information as mass/energy balances, operating conditions, such as temperature or pressure, reactions yield, and stoichiometry, as well as others (Hernández et al., 2014). The information and data required for the simulation of TiO_2_ nanoparticle production via green chemistry are taken from the literature and experimental results published by authors at lab-scale (Meramo-Hurtado et al., 2018). The application of exergy analysis and exergetic sensitivity analysis will provide insights into the implementation of this process at large scale for producing high-value nanomaterials.

### Exergy analysis of chemical processes

Exergy analysis is an important tool which can be used as an instrument to evaluate existing and emerging processing pathways from energy and thermodynamic viewpoints (Singh et al., 2019). Hamdy et al. (2019) applied an exergy-based method to quantify the effect of cold storage on thermodynamic performance of six liquefaction processes and identified the most cost-efficient process. In this study, exergy analysis was used as a decision-making tool for the selection of the best alternative for the liquefaction process. Zoder et al. (2018) developed a simulation and exergy analysis of energy conversion processes applied to gas and steam turbine cycles. The authors introduced a novel approach which combines process simulation, exergy analysis and object-oriented programming of a combined cycle, gas turbine system. In this case, exergy sensitivity analysis allowed identification of thermodynamic inefficiencies. Peters, Petrakopoulou & Dufour (2015) performed an exergy analysis to assess synthetic biofuel production via fast pyrolysis and hydrodeoxygenation upgrading. In such work, process simulation provided operating information to conduct exergy balances for each plant section and exergy inefficiencies were determined. These performance parameters revealed the potential for improvement of the process. Koç, Yağlı& Koç (2019) developed an exergy analysis of a subcritical/supercritical organic Rankine cycle for a gas heat recovery system. Exergy analysis was performed based on mass, energy and general exergy balance according to the net power production and energy/exergy inlet flows. Peralta-Ruiz, González-Delgado & Kafarov (2013) applied exergy analysis for screening process alternatives in microalgae oil extraction. The authors considered exergy analysis as a selection criteria for novel/emerging processing routes under sustainability targets. In the study, processing topologies were simulated using Aspen Plus^®^ software, and the exergy analyses were developed based on the extended mass and energy balances obtained from process simulation. Meramo-Hurtado, Ojeda-Delgado & Sánchez-Tuirán (2018) analyzed a bioethanol production plant design under exergy parameters considering rice residues as the main feedstock. Results showed that acid pretreatment was the stage with the lowest exergy efficiency. Bahadori & Nalbland Oshnuie (2019) developed an exergy analysis of indirect dimethyl production process to find the stages with low exergetic efficiencies. All of these contributions to development of the exergy concept show the relevance of the application of exergy analysis as a useful process evaluation tool.

## Materials & Methods

**Figure 1:**
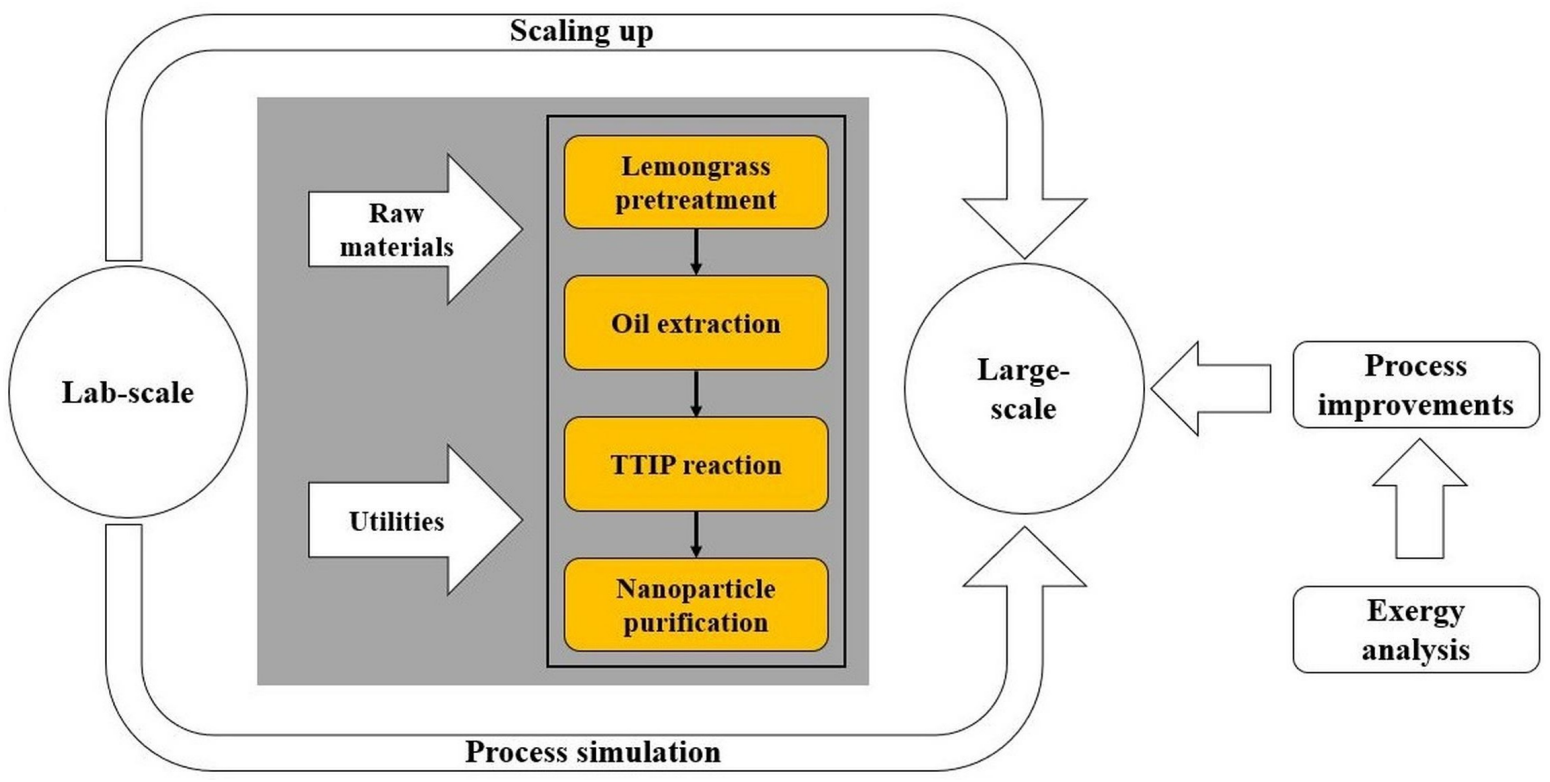
Scheme of the methodology to assess synthesis of TiO_2_ nanoparticles via green chemistry.

This section covers two main steps: (i) process simulation using Aspen Plus^®^, and (ii) mathematical modeling of exergy analysis around the system. Process simulation required setting an adequate thermodynamic model, which allows one to accurately estimate the physical-chemical properties of the process substances and compounds involved. The exergy assessment was performed according to the formulation of exergy analysis proposed by Peralta-Ruiz, González-Delgado & Kafarov (2013). Figure 1 depicts a schematic representation of the methodology followed to evaluate the greener production of TiO_2_ nanoparticles, in which, titanium isopropoxide (TTIP) and lemongrass are used as raw materials.

### Process simulation

The plant size was fixed according to the availability of raw materials, mainly, lemongrass. After selecting the production capacity of the plant, process simulation was performed based on the following steps:

I. Select chemical components of the process and operations.

II. Choose a proper thermodynamic model and state equation.

III. Set processing capacity.

IV. Set up input parameters as mass/energy flow rates, temperature, pressure, and stoichiometry of the reactions (Xuan, Lim & Yeo, 2014).

**Figure 2:**
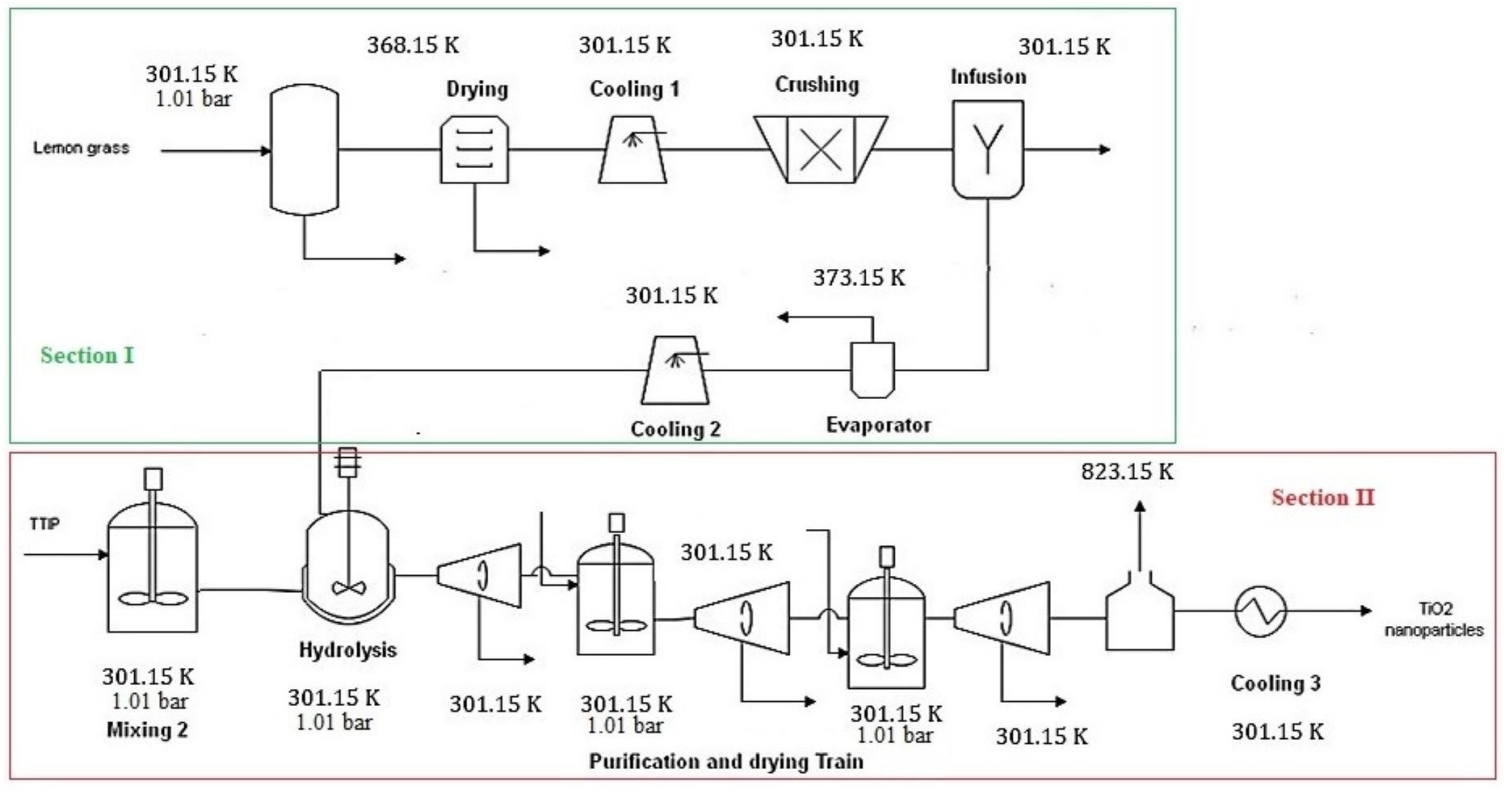
Process diagram of a large scale TiO_2_ nanoparticles production.

The commercial simulation software Aspen Plus^®^ was used to model and simulate the large-scale production process of TiO_2_ nanoparticles. Process boundaries were defined by the highlighted sections in red and green shown in Fig. 2. Several contributions use this tool to model existing and emerging technologies for chemical processing, which derived from a wide variety of feedstocks (e.g., biomass), processing routes and products (Meramo-Hurtado, Ojeda-Delgado & Sanchez-Tuiran, 2018; Ojeda et al., 2011b; Luo, Van der Voet & Huppes, 2010). The software Aspen Plus^®^ is characterized by an extensive, flexible and trusted property database containing a vast property collection of many compounds. Hence, most compounds involved in TiO_2_ nanoparticle synthesis were available in the database. Components unavailable in the software were created using the Molecule Editor in Aspen Plus^®^ and the physicochemical properties reported in the literature (Wooley & Putsche, 1996). This software also allows one to introduce many known constant properties and temperature-related parameters such as normal boiling point, Gibbs free energy of formation, critical properties, and acentric factors. In this work, it was necessary to create a user-defined component such as titanium isopropoxide. The Non-Random Two Liquids (NRTL) solution model was chosen as the thermodynamic-based model for process simulation because of its accuracy to predict properties of polar/non-polar mixtures.

### Process description

The large-scale process was designed for a capacity of 5,724.16 t/y of TiO_2_ nanoparticles because of the availability of raw materials. In Colombia, lemongrass production is the limiting factor on TiO_2_ nanoparticles synthesis with a national production above 30,000 t/y over the two last decades (Espinal et al., 2000). The extract of lemongrass (lemongrass oil) was used as a surfactant to ensure nanosized particles. Titanium isopropoxide (TTIP) was employed as a precursor of titanium in the photocatalytic product. The green synthesis process was divided into two main sections as shown in Fig. 2:

I Lemongrass oil extraction.
II Synthesis of TiO_2_ nanoparticles.

In the first section, lemongrass is pretreated to remove cellulosic material that may reduce the oil extraction yield. Then, dried lemongrass is cooled to room temperature (28 °C) and sent into a crushing unit for particle size reduction. An infusion-evaporation unit was used as a solid–liquid extraction method to collect oil extract with low moisture content. The oil extract is mainly composed of myrcene, neral, geraniol, citral and nerol, with a total oil composition of 1.10 wt% (Meramo-Hurtado et al., 2018). This process stream contains 7,284.31 t/y of phytochemicals (myrcene, neral, citral, among others) and an oil composition of 4.2 wt%. The second section involves a hydrolysis reaction to form nanoparticles. For simulation purposes, kinetics are significant parameters for reactor modeling (Sun et al., 2019). The general reaction mechanisms of the sol–gel synthesis of TiO_2_ from titanium alkoxide are shown as follows:

(1) \begin{eqnarray*}\mathrm{Ti}{ \left( \mathrm{OR} \right) }_{4}+4{\mathrm{H}}_{2}\mathrm{O}\rightarrow \mathrm{Ti}{ \left( \mathrm{OH} \right) }_{4}+4\mathrm{ROH}\end{eqnarray*}

(2) \begin{eqnarray*}\mathrm{Ti}{ \left( \mathrm{OH} \right) }_{4}\rightarrow \mathrm{Ti}{\mathrm{O}}_{2}+2{\mathrm{H}}_{2}\mathrm{O}\end{eqnarray*}

In the presence of water, titanium alkoxide is hydrolyzed (hydrolysis reaction) and then, condensation takes place to form an oxide network (Mahshid, Askari & Ghamsari, 2007; Gupta & Tripathi, 2012). The Arrhenius rate constants for TiO_2_ nanoparticle synthesis were reported by Oskam et al. (2003) with values ranged 10^−2^-10^−3^ nm^3^s^−1^ for 1/temperature between 0.002–0.0024 K^−1^. The overall reaction using TTIP as precursor is given by Nour, Sulaiman & Nour (2014):

(3) \begin{eqnarray*}\mathrm{Ti}{ \left( \mathrm{O}{\mathrm{C}}_{3}{\mathrm{H}}_{7} \right) }_{4}+2{\mathrm{H}}_{2}\mathrm{O}\rightarrow \mathrm{Ti}{\mathrm{O}}_{2}+4{\mathrm{C}}_{3}{\mathrm{H}}_{7}\mathrm{OH}.\end{eqnarray*}

After mixing the titanium precursor with water, lemongrass oil was added into the hydrolysis reactor. At the lab-scale, previous authors reported a conversion yield of 0.93 mol TTIP/mol TiO_2_ (Nour, Sulaiman & Nour, 2014). The resulting nanoparticles were sent to repetitive cycles of centrifugation and washing to remove the residual water and ethanol. Finally, moisture content of high purity nanoparticles was reduced in the calcination stage.

### Mathematical formulation for exergy analysis

The exergy analysis provides key indicators to improve process performance such as exergy loss or irreversibilities, percentage of exergy loss and exergy efficiency, which can be calculated per stage or around the entire system. Assumptions reported by Moreno-Sader, Meramo-Hurtado & González-Delgado (2019) were considered in this work:

• The whole process was assumed to be steady state.
• Kinetic exergy and potential exergy were neglected.
• The temperature of reference was taken as 298 K.

**Figure 3:**
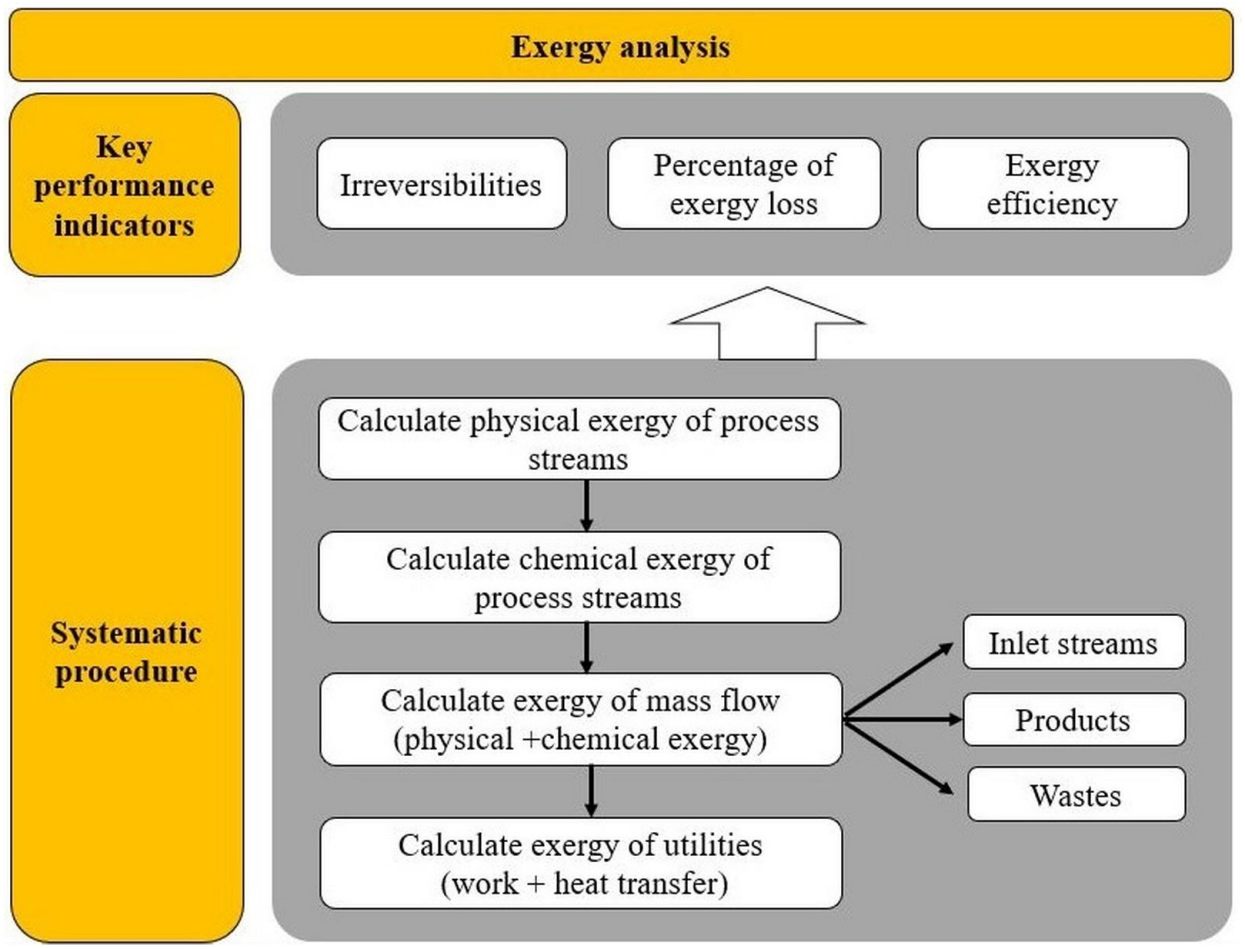
Scheme of procedure for exergy analysis.

The performance indicators from exergy analysis are functions of variables such as exergy of work, exergy due to heat transfer or exergy of the process stream. The calculations for exergy analysis are based on the research conducted by Yan et al. (2019), Yang et al. (2019b) and Yang et al. (2019a). For a better understanding of this section, a systematic procedure for exergy-based indicators is presented in Fig. 3.

As a first approach, the composition of process streams was needed to identify the main properties of components. The following equation was used to calculate physical exergy of process streams, where H and S, are the stream enthalpy and entropy, respectively.

(4) \begin{eqnarray*}{\mathrm{Ex}}_{\mathrm{p}}=\mathrm{H}-{\mathrm{H}}_{\mathrm{o}}-{\mathrm{T}}_{0} \left( \mathrm{S}-{\mathrm{S}}_{0} \right) \end{eqnarray*}

The second step is to calculate the specific chemical exergies of compounds, which are usually reported in the literature. This parameter is defined as the work that can be obtained when a substance reaches thermodynamic equilibrium through chemical reactions and is given by Eq. (5).

(5) \begin{eqnarray*}{\mathrm{Ex}}_{\mathrm{che}, \mathrm{i}}=\Delta {\mathrm{G}}_{\mathrm{f}}^{0}+\sum _{\mathrm{j}}{\mathrm{v}}_{\mathrm{j}}{\mathrm{Ex}}_{\mathrm{Ch}-\mathrm{j}}^{0}\end{eqnarray*}

where $\Delta {\mathrm{G}}_{\mathrm{f}}^{0}$ is the standard free energy of formation, ${\mathrm{Ex}}_{\mathrm{Ch}-\mathrm{j}}^{0}$ is the standard chemical exergy for element j, v_j_ is the number of atoms of j. The chemical exergy of process stream, i.e., a mixture (Ex_che_, depends on the chemical exergy of its components and is given by Eq. (6).

(6) \begin{eqnarray*}{\mathrm{Ex}}_{\mathrm{che}}=\sum {\mathrm{y}}_{\mathrm{i}}\ast {\mathrm{Ex}}_{\mathrm{ch}-\mathrm{i}}+\mathrm{R}{\mathrm{T}}_{0}\sum {\mathrm{y}}_{\mathrm{i}}\ast \ln \nolimits {\mathrm{y}}_{\mathrm{i}}\end{eqnarray*}

where R is the universal gas constant and y_i_ is the mole fraction of component i.

The following step is to estimate the exergy of mass flow by Eq. (7), as the sum of chemical exergy, physical exergy, potential exergy and kinetic exergy.

(7) \begin{eqnarray*}\mathrm{E}{\mathrm{x}}_{\text{mass}}={\mathrm{Ex}}_{\mathrm{che}}+{\mathrm{Ex}}_{\mathrm{p}}+{\mathrm{Ex}}_{\text{potential}}+{\mathrm{Ex}}_{\mathrm{kin}}\end{eqnarray*}

Kinetic and potential exergies are neglected in most cases because of their low values compared to physical and chemical exergies; hence, Eq. (7) can be simplified into Eq. (8).

(8) \begin{eqnarray*}{\mathrm{Ex}}_{\text{mass}}={\mathrm{Ex}}_{\mathrm{p}}+{\mathrm{Ex}}_{\mathrm{che}}\end{eqnarray*}

The exergy of utilities involves both exergy of work (Ex_work_) and exergy of heat stream (Ex_Q_) that are calculated through Eq. (9) and 10, respectively. Exergy for work is equal to work (W), as long as there are no changes in volume. Exergy by heat transfer depends on the temperature of the stream and the environmental temperature (taken as 298 K and 1 atm).

(9) \begin{eqnarray*}{\mathrm{Ex}}_{\text{work}}=\dot {W}\end{eqnarray*}

(10) \begin{eqnarray*}{\mathrm{Ex}}_{\mathrm{Q}}= \left( 1- \frac{{T}_{o}}{T} \right) \dot {Q}\end{eqnarray*}

where *Q* is the heat transfer rate, *T* is the system temperature, and *T*_*o*_ is the environmental temperature. Then the total exergy (Ex_total−in_ entering into the system is estimated from the results of exergy of streams and industrial services (Ojeda et al., 2011a). This parameter is given by Eq. (11).

(11) \begin{eqnarray*}{\mathrm{Ex}}_{\text{total}-\mathrm{in}}=\sum {\mathrm{Ex}}_{\text{mass}-\mathrm{in}}+\sum {\mathrm{Ex}}_{\text{utilities}-\mathrm{in}}\end{eqnarray*}

Where ∑Ex_mass−in_ is the exergy of all streams entering the system and ∑Ex_utilities−in_ is the total exergy of the utilities.

Exergy can leave a system by products’ and residues’ streams. This parameter is estimated by Eq. (12), where Ex_total−out_ is the total output exergy flow, ∑Ex_products−out_ is the total exergy of product streams, and ∑Ex_residues−out_ is the total exergy flow by the process wastes.

(12) \begin{eqnarray*}{\mathrm{Ex}}_{\text{total}-\mathrm{out}}=\sum {\mathrm{Ex}}_{\text{products}-\mathrm{out}}+\sum {\mathrm{Ex}}_{\text{residues}-\mathrm{out}}\end{eqnarray*}

Process irreversibilities Ex_*loss*_) is the exergy loss, i.e., the work not used through the process, and it is given by Eq. (13).

(13) \begin{eqnarray*}{\mathrm{Ex}}_{loss}=\sum {\mathrm{Ex}}_{\text{total}-\mathrm{in}}+\sum {\mathrm{Ex}}_{\text{products}-\mathrm{out}}\end{eqnarray*}

To identify the sources of highest losses within the process, the percentage of exergy loss (% Ex_loss_) in process stage *k* is calculated by Eq. (14).

(14) \begin{eqnarray*}\text{% Ex}{ }_{\text{loss}}= \left( \frac{\mathrm{Ex}{ }_{\text{loss},\mathrm{k}}}{\mathrm{Ex}{ }_{\text{total loss}}} \right) \ast 100\end{eqnarray*}

The global exergy efficiency (*η*_exergy_) indicates the profitability and effectiveness of the process at the industrial level. This performance indicator is given by Eq. (15).

(15) \begin{eqnarray*}{\eta }_{\text{exergy}}= \left( 1- \left( \frac{\mathrm{Ex}{ }_{\text{loss}}}{{\mathrm{Ex}}_{\text{total}-\mathrm{in}}} \right) \right) \ast 100\end{eqnarray*}

## Results

### Process simulation

The process simulation of the large-scale synthesis of TiO_2_ nanoparticles via green chemistry was performed under defined assumptions as follows:

• Process simulation was carried out at steady state with fixed conditions such as processing capacity, pressure of process stages set in 1.01 bar, room temperature at 301.15 K. The detailed operating conditions are summarized in Table 1.
• The hydrolysis reactor was simulated using a RStoich model for a defined conversion yield of 0.93 mol TTIP/mol TiO_2_.
• The centrifuge was modeled as a membrane to retain solid nanoparticles and the permeate was mainly a mixture of residual water and ethanol.
• The pump was assumed as part of the mixing stage to facilitate calculations
• Manipulator blocks were employed for washing, infusion and calcination stages.

The stream properties were estimated by a non-random two liquid (NRTL) thermodynamic model due to its well-known accuracy for this type of mixture. However, more detailed validation of the selected model is summarized in Table 2 by comparing the chemical properties of TiO_2_ nanoparticles provided by Aspen Plus^®^ with those reported in literature (Shi et al., 2013).

**Table 1:** Operating conditions for process stages of TiO_2_ nanoparticles production.

**Unit**	**Pressure (bar)**	**T(K)**
Cleaning	1.01	301.15
Washing	1.01	301.15
Drying	1.01	368.15
Infusion	1.01	301.15
Evaporation	1.01	373.15
Hydrolysis reactor	1.01	301.15
Centrifugation (1, 2 and 3)	1.01	301.15
Calcination	1.01	823.15

**Table 2:** Chemical properties of TiO_2_ nanoparticles provided by Aspen Plus software.

**Property**	This work	Shi et al. (2013)	Accuracy (%)
**Relative density (g/cm**^**3**^**)**	4.26	4.26	99.99%
**Molecular weight (g/mol)**	79.87	79.90	99.96%
**Boiling point (°C)**	2749.85	2972.00	92.52%
**Melting point (°C)**	1856.85	1843.00	99.24%

**Figure 4:**
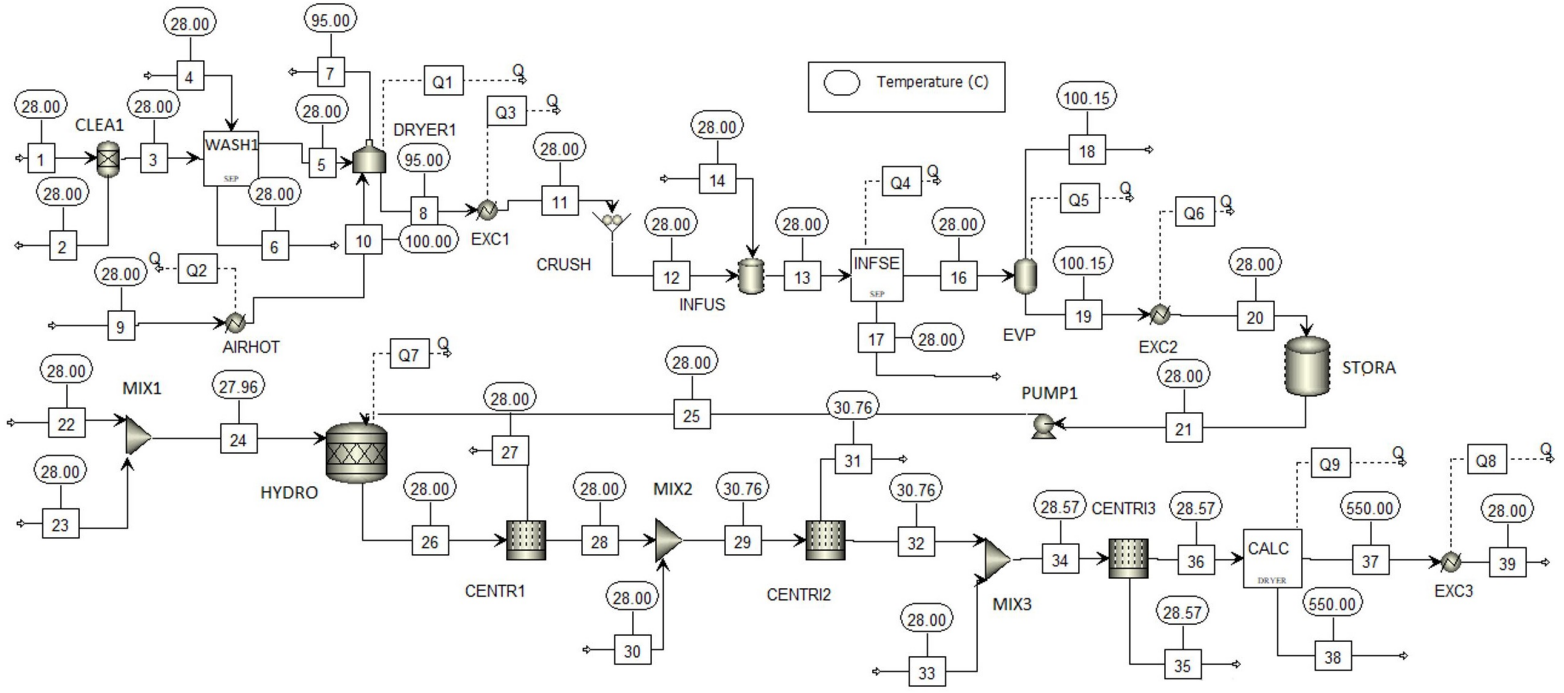
Process flowsheet of Large-scale production of TiO_2_ nanoparticles via green chemistry.

Figure 4 shows the process flowsheet for large-scale production of TiO_2_ nanoparticles via green chemistry. In first section, the STREAM 1 is the lemongrass stream used as raw material for oil extraction. Such stream is sent into a cleaning (CLEAN), washing (LAV1) and drying (DRYER 1) stages. After feedstock pretreatment, resulting stream (STREAM 11) passed through a crushing (CRUSH) stage to reduce size of particles and increase surface area. Then, liquid–solid extraction (INFUS) was carried out, followed by an evaporation (EVP) stage. The oil extract is collected, cooled until room temperature and stored with 96% wt. moisture content (STORA). The second section was simulated using models for reactors, mixer and centrifuge in Aspen Plus^®^ library. First, TTIP precursor is mixed with water (MIX 1) and the resulting mixture is fed into a hydrolysis reactor (HYDRO) along with the oil extract. The product stream is mainly nanoparticles (STREAM 26) requiring further purification. To this end, three centrifuges (CENTR1, CENTR2 and CENTR3) and one dryer (CALC) were employed to achieve high purity. Finally, TiO_2_ nanoparticles were cooled to room temperature (STREAM 39). Table 3 lists mass flowrates for main process streams of the simulated route. For a processing capacity of 32,675 t/y lemongrass and 5,724 t/y TTIP, simulation reported a production rate of 1,496 t/y TiO_2_ nanoparticles. Hence, the overall production yield was calculated in 0.26 kg TiO_2_/kg TTIP for greener synthesis of titanium-derived nanoparticles.

**Table 3:** Main process streams for production of TiO_2_ nanoparticles via Green Chemistry.

**Streams**	**1**	**19**	**22**	**39**
Temperature (K)	301.15	373.15	301.15	301.15
Pressure (kPa)	101.32	101.32	101.32	101.32
Mass Flow (kg/h)				
B-myrcene	2.04	0.47	0.00	0.00
3-Undecyne	1.19	0.55	0.00	0.00
Gerenial	27.10	22.46	0.00	0.00
Nerol	1.28	1.086	0.00	0.00
Cellulose	1882.97	0.00	0.00	0.00
Water	1466.9	608.58	0.00	0.00
TTIP	0.00	0.00	592.90	0.00
TiO_2_	0.00	0.00	0.00	154.84
NaOH	0.00	0.00	0.00	0.00
Total	3381.47	633.15	592.90	154.84

### Exergy analysis results

According to the well-defined methodology for exergy analysis, components of process streams were identified in order to search their specific chemical exergies. Open literature layout an extensive database of chemical exergy, e.g., Bilgen, Keles & Kaygusuz (2012) reported values of chemical exergy for many solvents and bio-oils. In this work, the standard chemical exergies reported by Rivero & Garfias (2006) and Szargut (2007) were employed to perform exergy analysis. Table 4 shows the specific chemical exergy for myrcene, uncedyne, nerol, cellulose, water, oxygen, TTIP, TiO_2_, among others, which are the main components of the oil extract, precursor and nanoparticles.

**Table 4:** Specific chemical exergies of substances involved in the process.

**Component**	**Chemical Exergy (kJ/kg)**
Myrcene	70983.63
Undecyne	74842.56
Gerenial	63771.67
Nerol	67086.54
Cellulose	18807.95
Water	42.77
Oxygen	330.83
Nitrogen	51.43
TTIP	52420.17
TiO_2_	263.81
Propanol	33396.66
Etanol	29497.00

The thermodynamic properties of specific chemical exergy, enthalpy and entropy were key data to continue with the next step in exergy analysis. These properties’ values were entered into Eqs. (2) and (4) to calculate the physical and chemical exergy of process streams, respectively. Table 5 summarizes the results obtained for the main process streams including raw materials and product.

**Table 5:** Chemical exergy of main components for TiO_2_ nanoparticle production process.

**Stream**	**Physical Exergy flow (MJ/h)**	**Chemical exergy flow (MJ/h)**	**Mass flow (t/year)**
1	0.17	41155.07	32674.71
19	0.06	1764.77	6118.02
22	2.31	959.32	196584.00
39	0.001	45.02	1496.16

**Figure 5:**
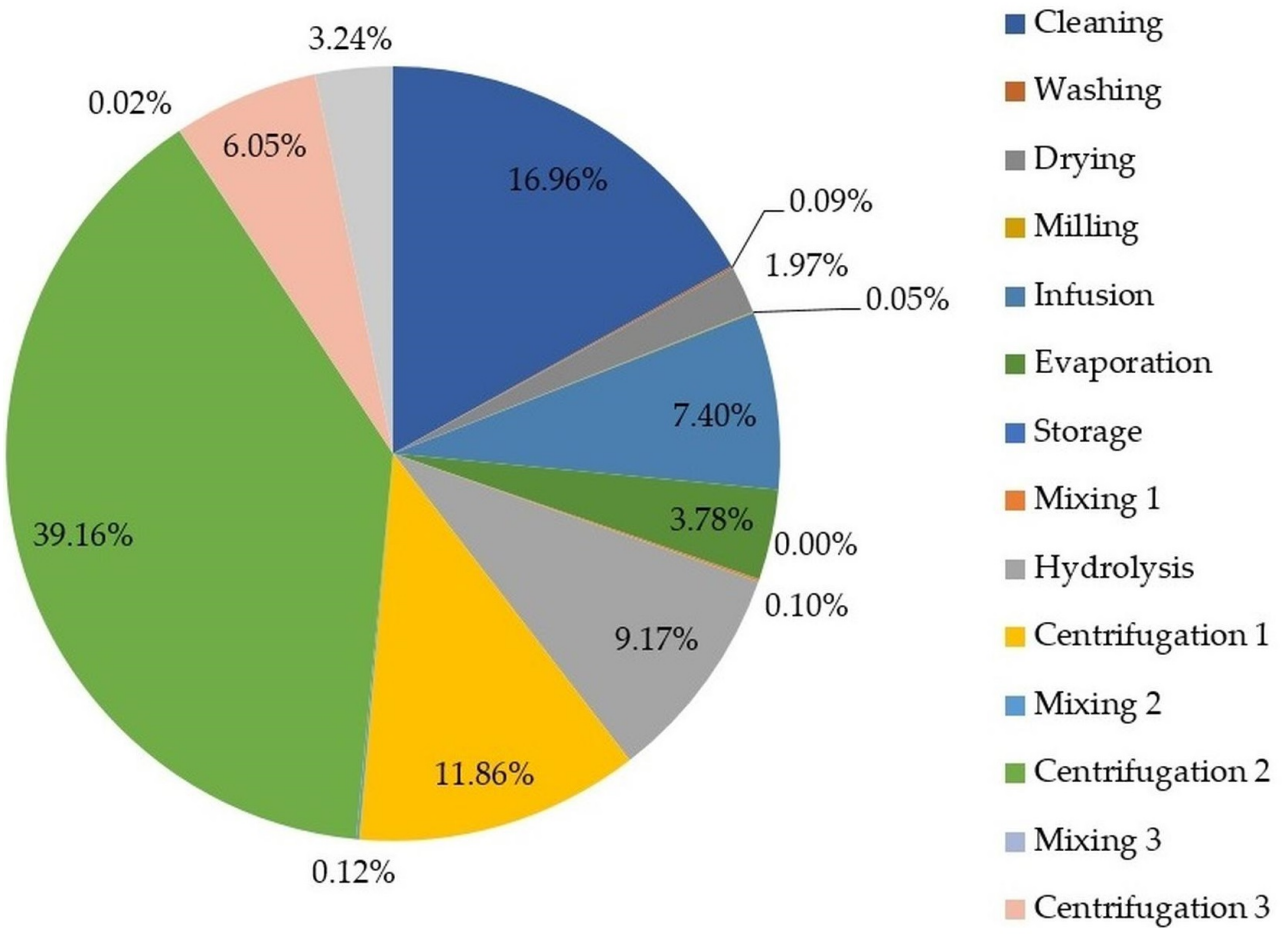
Percentage of exergy destruction by process stage.

Figure 5 depicts the contribution of process stages to exergy loss (or irreversibilities) to identify stages needing improvement from an energy viewpoint.

**Figure 6:**
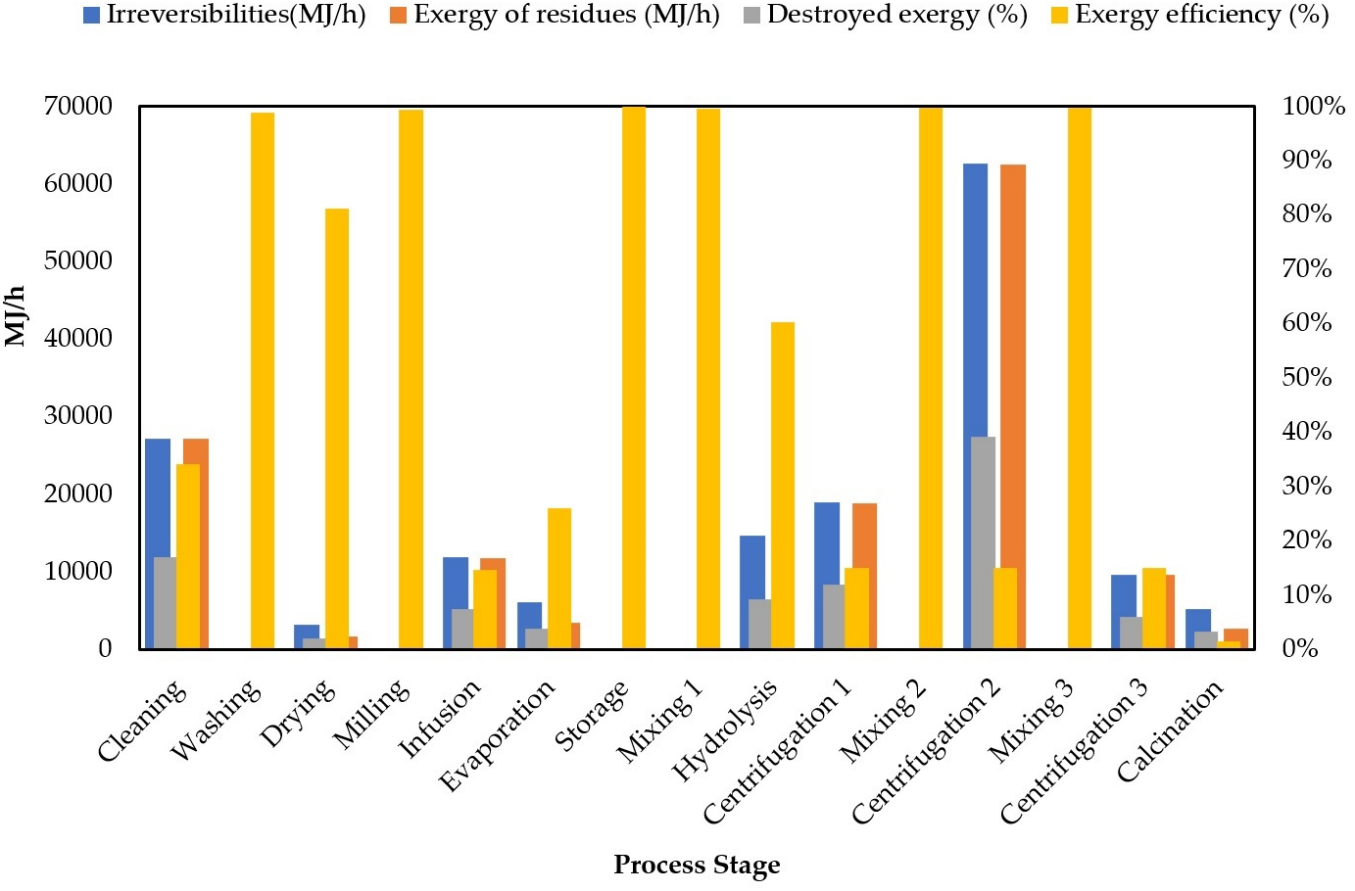
Comparison of exergy destruction for each processing route.

Figure 6 shows the exergy analysis results for each process stage reporting irreversibilities, exergy loss or destroyed exergy (%), exergy of residues and exergy efficiency.

**Figure 7:**
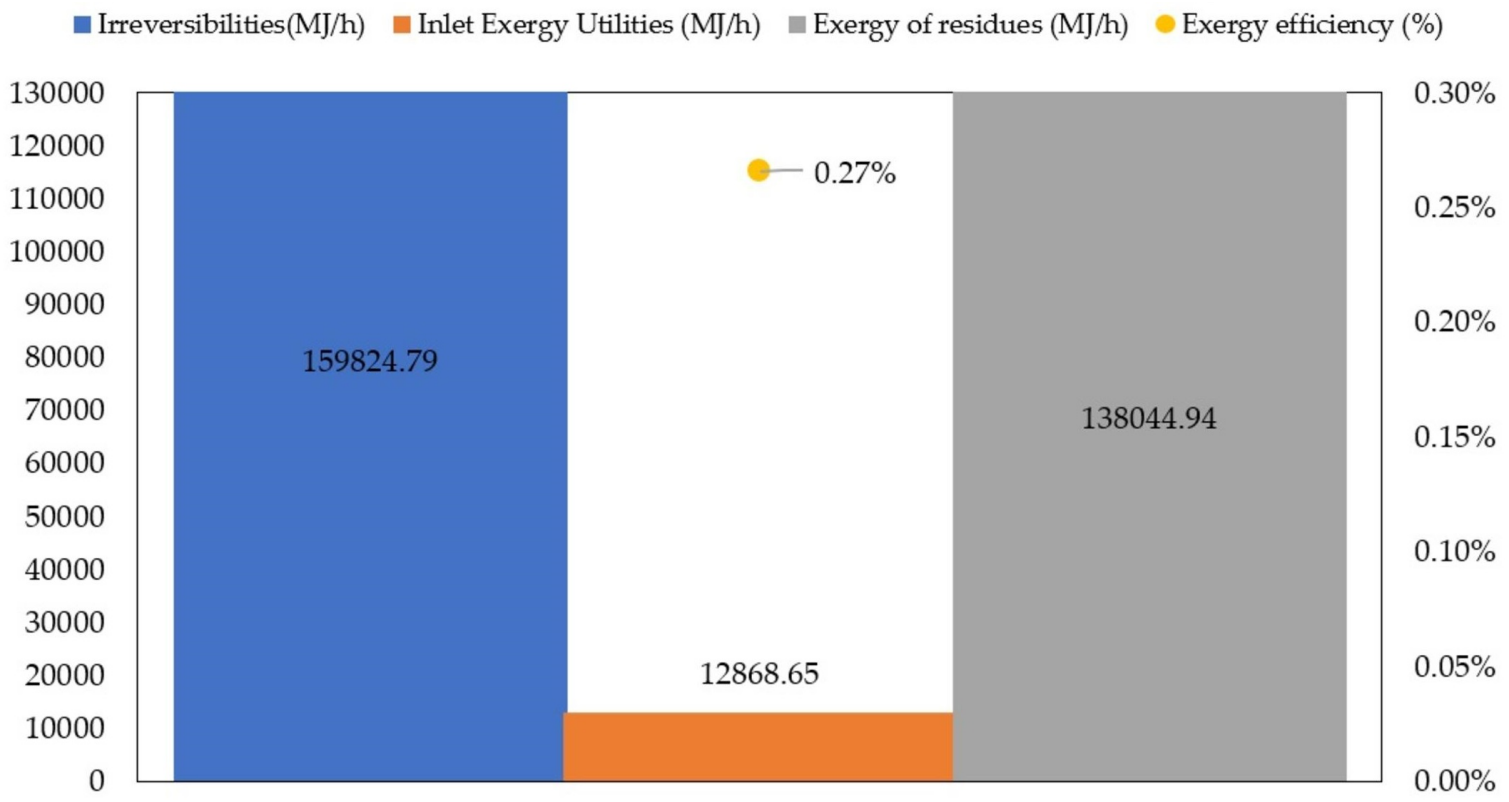
Overall exergetic performance of large-scale TiO_2_ production via green chemistry.

The overall energy performance of large-scale TiO_2_ production via green chemistry is shown in Fig. 7.

## Discussion

The process simulation provided extended mass and energy balances that are mandatory to analyze energetic performance under the exergy concept. Although the green chemistry synthesis may change physicochemical properties of the TiO_2_ nanoparticles, high accuracy (>90%) was achieved by the NRTL thermodynamic model compared with commercial nanoparticles (see Table 2).

The chemical exergies of compounds (see Table 4) provided insights about the useful energy contained within them. The highest values were reached by phytochemicals in the oil extract, followed by TTIP, ethanol and propanol. Thus, oil extract has more useful energy than other process substances. Table 5 reported higher chemical and physical exergies for lemongrass and titanium isopropoxide compared with titanium dioxide nanoparticles, which may be attributed to:

i) The useful energy contained in base components.
ii) The higher mass flowrate of raw materials multiplying specific exergy as conversion yield was 0.26 kg TiO_2_/kg TTIP.

These results suggested degradation of energy quality for chemical compounds within the green synthesis route. Therefore, a global idea of the low energy performance of large-scale TiO_2_ nanoparticles may be concluded from this analysis.

As shown in Fig. 5, the evaporation stage during oil extraction from lemongrass (section 1) contributed the most to exergy loss with 39.16%, which is explained by the high energy demand as well as the residual steam leaving the evaporator. Alternatives to reduce exergy loss of such stages must be addressed by reusing residual steam and varying operating conditions. Galovic & Marija (2009) studied the exergy destruction (i.e., exergy loss) of an evaporator considering temperature differences between streams as a key parameter. They found a continuous increase of exergy destruction with the increase of the ratio of input thermodynamic temperatures of process streams. Based on this, it is suggested to perform an exergy-based sensitivity analysis to determine the effect of varying this ratio on exergy loss. Both cleaning and centrifugation 1 stages also reported high contributions to exergy loss with 16.96% and 11.86%, respectively. These findings are explained by the huge amount of cellulosic wastes removed from lemongrass feedstock during the cleaning stage. Such exergy loss may be reduced by cellulosic waste valorization to generate energy within the process under a co-generation concept.

Centrifugations 1, 2, 3 and calcination stages are needed for nanoparticles purification (section II), however, they must be examined in detail to identify the reasons why these stages reached such low exergy efficiencies (see Fig. 6). Meramo-Hurtado, Herrera-Barros & Gonzalez-Delgado (2019) performed a comparative exergy assessment of chitosan microbeads modified with nanoparticles. They found the highest irreversibilities in the separation train after producing chitosan-TiO_2_ microbeads. The separation is composed of three consecutive centrifugations representing 53% of the total irreversibilities. Centrifugation stages during TiO_2_ nanoparticle preparation are low-efficiency due to the huge quantity of residues leaving the system from interstage mixing with ethanol and water. Calcination is also a high-energy consuming stage with residue generation at 550 °C. This temperature condition increases the exergy of heat flow in the overall exergy analysis. Centrifugation 2 and cleaning stages achieved the highest results for irreversibilities with 62,595.07 MJ/h and 27,107.17 MJ/h, respectively. The performance of such parameter is congruent with the exergy efficiency estimations for centrifugation 2. This value was expected for cleaning due to its contributions to exergy losses as previously reported. A similar tendency was also found for destroyed exergy (%) in both stages. The highest exergy of residues was reached by centrifugation 2 (62,459.83 MJ/h), followed by cleaning (27,224.14 MJ/h) and centrifugation 1 (18,862.93 MJ/h) stages. Process improvements to address reductions in exergy of residues lie in the valorization of wastes within process stages or optimization of the synthesis route to reduce mass and energy requirements. In general, many efforts must be made to overcome exergy limitations on the applications of green chemistry-based routes.

The total exergy of inlet utilities and total exergy of residues reported values of 12,868.65 MJ/h and 138,044.94 MJ/h, respectively. As shown in Fig. 7, the main contributors to the exergy of utilities were drying, calcination and cooling stages. Total irreversibilities were calculated as 15,9824.80 MJ/h. Grubb & Bakshi (2008) performed energy and environmental evaluation of conventional synthesis of TiO_2_ nanoparticles and reported an irreversibilities rate above 7,000 MJ/h for material exergy with the largest contributions from spray hydrolysis. Although these results are not directly comparable to each other, both works provide insights about exergy mapping of the main sources of irreversibilities. The overall exergy efficiency was estimated as 0.27%, a significantly low value for a chemical process. However, such results may be expected for emerging technologies such as the green synthesis route because of the unknown behavior at large-scale (Meramo-Hurtado et al., 2018). To increase overall exergy efficiency, a wide range of alternatives can be considered as key process improvements. For example, the application of methodologies for process optimization such as mass or energy integration (El-Halwagi, 2012), or the development of process intensification strategies (Ma et al., 2018).

## Conclusions

In this work, an exergy analysis was developed for a large-scale TiO_2_ production via green chemistry to identify potential improvement opportunities based on an energy viewpoint. It is important to highlight that this first approach to a large-scale, production route under green chemistry concepts was developed to synthesize a green catalyst which can be employed for wastewater treatment systems. Results revealed that this process is thermodynamically inefficient with a global exergetic efficiency of 0.27%. The calcination stage showed the lowest exergy efficiency because of the heat requirements for performing this operation. Also, large quantities of residues (10,354.76 t/y) at high temperature (550 °C) are associated with this stage. In the cleaning stage, significant quantities of exergy are lost as outlet waste, so it is recommended to add a co-generation system to avoid exergy losses. Finally, it is recommended that future works incorporate process integration or intensification strategies in order to obtain the most suitable design in terms of energy.

##  Supplemental Information

10.7717/peerj.8113/supp-1 Data S1 Raw data

10.7717/peerj.8113/supp-2 Data S2 Simulation data

## Additional Information and Declarations

### Data Availability

The following information was supplied regarding data availability: The raw data for exergy analysis and simulation are available in the Supplemental Files.

## References

[ref-1] Acosta-Herazo R, Ángel M, Li G, Machuca-Martínez F (2019). Impact of photocatalyst optical properties on the efficiency of solar photocatalytic reactors rationalized by the concepts of initial rate of photon absorption (IRPA) dimensionless boundary layer of photon absorption and apparent optical thickness. Chemical Engineering Journal.

[ref-2] Anastas P, Warner J (1998). Green chemistry: theory and practice.

[ref-3] Arya A, Mishra V, Chundawat TS (2019). Green synthesis of silver nanoparticles from green algae (Botryococcus braunii) and its catalytic behavior for the synthesis of benzimidazoles. Chemical Data Collections.

[ref-4] Bahadori F, Nalbland Oshnuie M (2019). Exergy analysis of indirect dimethyl ether production process. Sustainable Energy Technologies and Assessments.

[ref-5] Bhojwani S, Topolski K, Mukherjee R, Sengupta D, El-Halwagi MM (2019). Technology review and data analysis for cost assessment of water treatment systems. Science of the Total Environment.

[ref-6] Bilgen S, Keles S, Kaygusuz K (2012). Calculation of higher and lower heating values and chemical exergy values of liquid products obtained from pyrolysis of hazelnut cupulae. Energy.

[ref-7] Bustillo-Lecompte CF, Kakar D, Mehrvar M (2018). Photochemical treatment of benzene, toluene, ethylbenzene, and xylenes (BTEX) in aqueous solutions using advanced oxidation processes: towards a cleaner production in the petroleum refining and petrochemical industries. Journal of Cleaner Production.

[ref-8] Deyris P-A, Grison C (2018). Nature, ecology and chemistry: an unusual combination for a new green catalysis, ecocatalysis. Current Opinion in Green and Sustainable Chemistry.

[ref-9] El-Halwagi MM (2012). Sustainable design through process integration. Fundamentals and applications to industrial pollution prevention, resource conservation, and profitability enhancement.

[ref-10] Espinal S, Garcia N, Garzón M, Gomez F, Jaller S, Medina J, Moreno Z, Pardo O, Repetto E, Ruiz M del P, Saenz P, Torres MC, Torres A, Villareal A (2000). Limas y limones. Inteligencia de Mercados: Perfil de Producto.

[ref-11] Galovic A, Marija Z (2009). Analysis of exergy destruction of an evaporator or/and a condenser. Strojarstvo.

[ref-12] Grubb GF, Bakshi BR (2008). Energetic and environmental evaluation of titanium dioxide nanoparticles. IEEE International Symposium on Electronics and the Environment.

[ref-13] Gupta SM, Tripathi M (2012). A review on the synthesis of TiO_2_ nanoparticles by solution route. Central European Journal of Chemistry.

[ref-14] Haider AJ, Al-Anbari RH, Kadhim GR, Salame CT (2017). Exploring potential environmental applications of TiO_2_ Nanoparticles. Energy Procedia.

[ref-15] Hamdy S, Moser F, Morosuk T, Tsatsaronis G (2019). Exergy-based and economic evaluation of liquefaction processes for cryogenics energy storage. Energies.

[ref-16] Hendershot DC (2015). Green chemistry and process safety. Journal of Chemical Health and Safety.

[ref-17] Hernández V, Romero-García JM, Dávila JA, Castro E, Cardona CA (2014). Techno-economic and environmental assessment of an olive stone based biorefinery. Resources, Conservation and Recycling.

[ref-18] Karpagavinayagam P, Vedhi C (2019). Green synthesis of iron oxide nanoparticles using *Avicennia marina* flower extract. Vacuum.

[ref-19] Koç Y, Yağlı H, Koç A (2019). Exergy analysis and performance improvement of a subcritical/supercritical organic rankine cycle (ORC) for exhaust gas waste heat recovery in a biogas fuelled combined heat and power (CHP) engine through the use of regeneration. Energies.

[ref-20] Luo L, Van der Voet E, Huppes G (2010). Biorefining of lignocellulosic feedstock—technical, economic and environmental considerations. Bioresource Technology.

[ref-21] Ma Y, Zhang X, Zhu Z, Wang Y, Gao J, Cui P (2018). Process intensification and waste minimization for ibuprofen synthesis process. Journal of Cleaner Production.

[ref-22] Mahshid S, Askari M, Ghamsari MS (2007). Synthesis of TiO_2_ nanoparticles by hydrolysis and peptization of titanium isopropoxide solution. Journal of Materials Processing Technology.

[ref-23] Martínez González A, Silva Lora EE, Escobar Palacio JC (2019). Syngas production from oil sludge gasification and its potential use in power generation systems: an energy and exergy analysis. Energy.

[ref-24] Meramo-Hurtado S, Bonfante H, De Avila-Montiel G, Herrera-Barros A, Gonzalez-Delgado A (2018). Environmental assessment of a large-scale production of TiO_2_ nanoparticles via green chemistry. Chemical Engineering Transactions.

[ref-25] Meramo-Hurtado S, Herrera-Barros A, Gonzalez-Delgado Á (2019). Evaluation of large-scale production of chitosan microbeads modified with nanoparticles based on exergy analysis. Energies.

[ref-26] Meramo-Hurtado S, Ojeda-Delgado K, Sanchez-Tuiran E (2018). Computer-aided environmental assessment and energy integration of bioethanol production from rice residues. Contemporary Engineering Sciences.

[ref-27] Meramo-Hurtado S, Ojeda-Delgado K, Sánchez-Tuirán E (2018). Exergy analysis of bioethanol production from rice residues. Contemporary Engineering Sciences.

[ref-28] Moreno-Sader K, Meramo-Hurtado SI, González-Delgado AD (2019). Computer-aided environmental and exergy analysis as decision-making tools for selecting bio-oil feedstocks. Renewable and Sustainable Energy Reviews.

[ref-29] Nour AH, Sulaiman ZA, Nour AH (2014). Comparative study of lemongrass (Cymbopogon Citratus) essential oil extracted by microwave-assisted hydrodistillation (MAHD) and conventional hydrodistillation (HD) method. International Journal of Chemical Engineering and Applications.

[ref-30] Nwankwo U, Bucher R, Ekwealor ABC, Khamlich S, Maaza M, Ezema FI (2019). Synthesis and characterizations of rutile-TiO_2_ nanoparticles derived from chitin for potential photocatalytic applications. Vacuum.

[ref-31] Ojeda K, Sánchez E, El-Halwagi M, Kafarov V (2011a). Exergy analysis and process integration of bioethanol production from acid pre-treated biomass: comparison of SHF, SSF and SSCF pathways. Chemical Engineering Journal.

[ref-32] Ojeda K, Sánchez E, Suarez J, Avila O, Quintero V, El-Halwagi M, Kafarov V (2011b). Application of computer-aided process engineering and exergy analysis to evaluate different routes of biofuels production from lignocellulosic biomass. Industrial & Engineering Chemistry Research.

[ref-33] Oskam G, Nellore A, Penn RL, Searson PC (2003). The growth kinetics of TiO_2_ nanoparticles from Titanium (IV) Alkoxide at high water/ titanium ratio. The Journal of Physical Chemistry B.

[ref-34] Peralta-Ruiz Y, González-Delgado AD, Kafarov V (2013). Evaluation of alternatives for microalgae oil extraction based on exergy analysis. Applied Energy.

[ref-35] Peters J, Petrakopoulou F, Dufour J (2015). Exergy analysis of synthetic biofuel production via fast pyrolysis and hydroupgrading. Energy.

[ref-36] Querol E, Gonzalez-Regueral B, Perez-Benedito JL (2013). Practical approach to exergy and thermoeconomic analyses of industrial processes. Springer briefs in energy.

[ref-37] Rivero RÃ, Garfias M (2006). Standard chemical exergy of elements updated. Energy.

[ref-38] Shi H, Magaye R, Castranova V, Zhao J (2013). Titanium dioxide nanoparticles: a review of current toxicological data. Particle and Fibre Toxicology.

[ref-39] Singh G, Singh PJ, Tyagi VV, Barnwal P, Pandey AK (2019). Exergy and thermo-economic analysis of ghee production plant in dairy industry. Energy.

[ref-40] Sun S, Yang A, Chien I, Shen W, Wei S, Ren J, Zhang X (2019). Intensification and performance assessment for synthesis of 2-methoxy-2-methyl-heptane through the combined use of different pressure thermally coupled reactive distillation and heat integration technique. Chemical Engineering and Processing - Process Intensification.

[ref-41] Szargut J (2007). Egzergia. Poradnik obliczania I stosowania.

[ref-42] Wang P, Lombi E, Zhao FJ, Kopittke PM (2016). Nanotechnology: a new opportunity in plant sciences. Trends in Plant Science.

[ref-43] Wooley RJ, Putsche V (1996). Development of an ASPEN PLUS physical property database for biofuels components.

[ref-44] Xuan T, Lim Y, Yeo H (2014). Techno-economic analysis of biooil production process from palm empty fruit bunches. Energy Conversion and Management.

[ref-45] Yan C, Lv L, Eslamimanesh A, Shen W (2019). Application of retrofitted design and optimization framework based on the exergy analysis to a crude oil distillation plant. Applied Thermal Engineering.

[ref-46] Yang A, Jin S, Shen W, Cui P, Chien I, Ren J (2019a). Investigation of energy-saving azeotropic dividing wall column to achieve cleaner production via heat exchanger network and heat pump technique. Journal of Cleaner Production.

[ref-47] Yang A, Sun S, Shi T, Xu D, Ren J, Shen W, Sun S, Shi T, Xu D, Ren J, Shen W (2019b). Energy-efficient extractive pressure-swing distillation for separating binary minimum azeotropic mixture dimethyl carbonate and ethanol. Separation and Purification Technology.

[ref-48] Zoder M, Balke J, Hofmann M, Tsatsaronis G (2018). Simulation and exergy analysis of energy conversion processes using a free and open-source framework—python-based object-oriented programming for gas- and steam turbine cycles. Energies.

